# Effects of Simulated Acid Rain on Photosynthesis in *Pinus massoniana* and *Cunninghamia lanceolata* in Terms of Prompt Fluorescence, Delayed Fluorescence, and Modulated Reflection at 820 nm

**DOI:** 10.3390/plants13050622

**Published:** 2024-02-24

**Authors:** Pengzhou Shu, Xiaofei Gong, Yanlei Du, Yini Han, Songheng Jin, Zhongxu Wang, Penghong Qian, Xueqin Li

**Affiliations:** 1 Jiyang College, Zhejiang A&F University, Zhuji 311800, China; 13588357598@163.com (P.S.); han-yini@hotmail.com (Y.H.); shjin@zafu.edu.cn (S.J.); 17757522620@163.com (Z.W.); 13454793067@163.com (P.Q.); 2 Ecological Forestry Development Center of Suichang County, Lishui 323300, China; dhc200528@163.com; 3 Environmental Protection Monitoring Station of Changxing County, Huzhou 313000, China; duyanlei007@126.com

**Keywords:** simulated acid rain, chlorophyll a fluorescence induction kinetics, 820 nm transmission kinetics, JIP-test, delayed fluorescence, coniferous trees

## Abstract

The effects of simulated acid rain (SAR) on the photosynthetic performance of subtropical coniferous species have not been thoroughly investigated. In this study, we treated two coniferous species, *Pinus massoniana* (PM) and *Cunninghamia lanceolata* (CL), with four gradients of SAR and then analyzed their photosynthetic activities through measurements of gas exchange, prompt fluorescence (PF), delayed fluorescence (DF), and modulated reflection at 820 nm (MR_820_). Gas exchange analysis indicated that the decrease in the net photosynthetic rate (Pn) in PM and CL was unrelated to stomatal factors. For the PF transients, SAR induced positive K-band and L-band, a significant reduction in photosynthetic performance index (PI_ABS_), the quantum yield of electron transfer per unit cross-section (ET_O_/CS_m_), and maximal photochemical efficiency of photosystem II (F_v_/F_m_). Analysis of the MR_820_ kinetics showed that the re-reduction kinetics of PSI reaction center (P700^+^) and plastocyanin (PC^+^) became slower and occurred at later times under SAR treatment. For the DF signals, a decrease in the amplitude of the DF induction curve reduced the maximum value of DF (I_1_). These results suggested that SAR obstructed photosystem II (PSII) donor-side and acceptor-side electron transfer capacity, impaired the connectivity between PSII and PSI, and destroyed the oxygen-evolving complex (OEC). However, PM was better able to withstand SAR stress than CL, likely because of the activation of a protective mechanism.

## 1. Introduction

Acid rain has become one of the most serious environmental problems worldwide due to decades of industrial development as well as increases in energy demands and emissions of certain gases, including sulfur dioxide (SO_2_) and nitrogen oxides (NO_X_) [1]. In terms of the total area affected by acid rain, China ranks third behind Europe and North America [2,3]. The acid-rain-affected regions in China account for 3.8% of the total land area, with Zhejiang province among the regions most severely affected by acid rain (average acid rain rate of 47.9%) [4]. According to Li et al. [5], because of improvements in the energy supply structure (e.g., the use of renewable energy sources) and significant increases in NO_X_ emissions from vehicle exhausts, the acids associated with acid rain in the subtropical region have recently changed from mainly sulfuric acid to a mixture of sulfuric acid and nitric acid. Despite the active efforts of government departments to control acid rain and mitigate its adverse effects, it is still causing major environmental problems in the subtropical region.

Simulated acid rain (SAR) initially affects plants by damaging the leaf wax, which leads to the rupture of the epidermis [5]. Additionally, the outer stomatal wall on the abaxial side of leaves also ruptures in response to SAR [6]. The morphology of some epidermal cells is consistent with the effects of lysis [6]. Plants accumulate phenolic compounds in necrotic areas [7]. Prolonged exposure to low-pH stress can damage the leaf mesophyll, ultimately leading to the complete collapse of mesophyll cells [7]. Moreover, SAR can also alter the physical and chemical properties of soil, thereby affecting the absorption of water and nutrients by plant roots, inhibiting plant growth and development, exacerbating plant diseases and pest infestations, accelerating leaf yellowing and shedding, and leading to the severe degradation of terrestrial ecosystems [8]. In evergreen trees in the subtropical region, only low-pH acidic conditions have a significant effect, while a high pH does not [1,9]. Studies have been conducted on the effects of SAR on plants [6,10,11] and nutrient elements [12]. Recent studies have also explored how the frequency of SAR influences soil microorganisms and plants [13]. There has been considerable research on the effects of SAR on the morphological features and photosynthetic activities of plants. More specifically, the related research on economically important coniferous species, such as *Pinus massoniana* (PM) and *Cunninghamia lanceolata* (CL), treated with SAR has mainly focused on biological characteristics, including growth [11], physiological functions [14], and ecological functions [15,16].

In terms of plant physiology, subtropical evergreen broad-leaved trees are more tolerant to acidic conditions than coniferous trees [17]. This is in accordance with the results of an earlier study involving an examination of spectral reflectance, which indicated that coniferous tree species are more sensitive to SAR than broad-leaved tree species [1]. There are few reports describing the changes in the growth characteristics and photosynthetic performance of PM and CL in response to SAR. In addition to being the most representative coniferous tree species in the subtropical region, PM and CL are the most widely cultivated timber species in China [18].

The development of the Multi-Function Plant Efficiency Analyzer (M-PEA) for the simultaneous measurement of prompt chlorophyll a fluorescence (PF), delayed chlorophyll a fluorescence (DF), and modulated reflection (MR) has enabled the examination of the changes in forward and reverse electron transport and/or the redox state of photosystem I (PSI) in many plant species [19,20]. The correlations among the aforementioned signals can provide mutually corroborating and complementary insights into the photosynthetic electron transport chain, including the forward and reverse electron flow and the cyclic electron flow around PSI. A previous study verified the utility of PF, DF, and MR at 820 nm (MR_820_) curves for analyzing the degree of plant stress [21]. The PF increases in three phases (O–J, J–I, and I–P), which represent three distinct reduction reactions in the electron transport chain. During photosynthesis, PF is detectable after the dark-to-light transition, whereas DF can be detected during the light-to-dark transition [19]. In addition, DF is mainly emitted by photosystem II (PSII) rather than PS I [22]. Based on this energy cascade, the PF emitted by chlorophyll a reflects photosynthetic vitality, especially for PSII, and can be measured under saturated actinic light at a wavelength of 627 ± 10 nm [23]. The transient MR signals at 820 nm suggest that the transport of electrons beyond the plastoquinone (PQ) pool in PSI can be measured under modulated light at a wavelength of 820 ± 25 nm. The DF denoting the charge recombination and re-population of excited PSII antenna chlorophyll can be measured under far-red light at a wavelength of 735 ± 15 nm [18,24].

To date, PF, DF, and the MR_820_ curve have been widely used during analyses of the effects of heat stress [25], salt stress [26], drought stress [27], and heavy metal stress [28]. However, there have been relatively few studies that combined PF, DF, the MR_820_ curve, and JIP test parameters to investigate the mechanism underlying the response of coniferous tree species to SAR. The objectives of this study were to use the above-mentioned methods to elucidate the mechanism mediating the response of subtropical coniferous species to SAR and to reveal the diversity in these responses, thereby providing a theoretical basis for the development of effective methods for protecting subtropical coniferous species from acid rain.

## 2. Results

**Figure 1:**
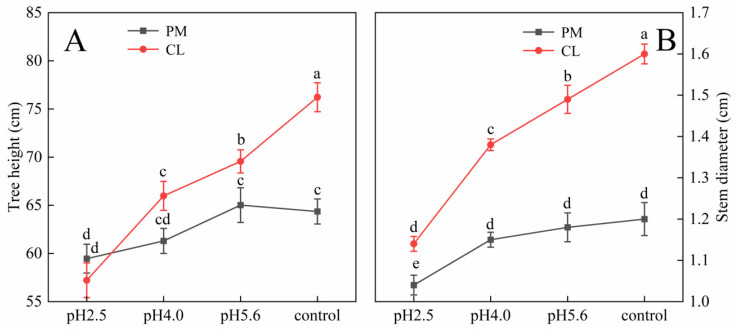
Effects of SAR stress on tree height (**A**) and stem diameter (**B**) of PM and CL. Each value is the mean ± SE (n = 3). Different lowercase letters indicates a significant difference at *p* < 0.05. The gas exchange parameters of PM and CL following the SAR treatments are presented in Figure 2. The application of the SAR solutions significantly decreased Pn and Gs for both PM and CL, whereas Ci increased as the SAR stress increased. After the SAR treatments, Pn and Gs were higher for PM than for CL, whereas the opposite trend was observed for Ci. Compared with the effects of the control treatment, the SAR pH 2.5 treatment of PM and CL resulted in decreases in Pn (by 82.04% and 84.14%, respectively) and Gs (by 81.10% and 84.88%, respectively), but increases in Ci (by 36.02% and 40.37%, respectively).

The SAR treatment inhibited PM and CL growth (Figure 1). Decreases in the pH of the SAR solution resulted in decreases in the tree height and stem diameter of both species. At pH 4.0, the tree height of CL decreased more than that of PM. Additionally, the stem diameter of CL decreased more significantly than that of PM at pH 2.5.

**Figure 2:**
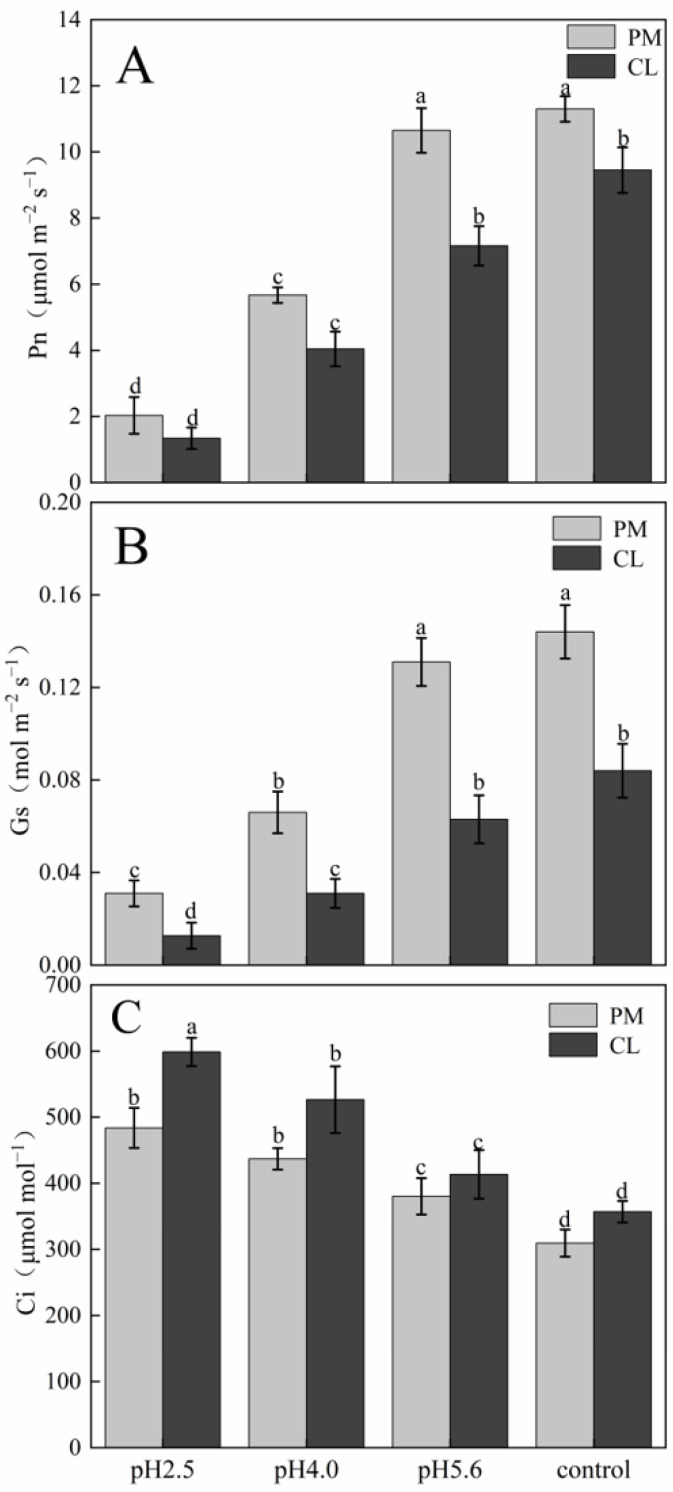
Effects of SAR on gas exchange in PM and CL: (**A**) Net photosynthetic rate (Pn). (**B**) Stomatal conductance (Gs). (**C**) Intercellular CO_2_ concentration (Ci). Each value is the mean ± SE (n = 3). Different lowercase letters indicates a significant difference at *p* < 0.05. The photosynthetic pigment contents in PM and CL decreased as the pH of the SAR solution decreased. Moreover, there was an inverse correlation between the photosynthetic pigment content and the SAR stress level (Figure 3). At pH 2.5, the chlorophyll a contents of PM and CL decreased by 49.00% and 89.04%, respectively. The chlorophyll b contents of PM and CL decreased by 54.74% and 82.20%, respectively. The total chlorophyll contents of PM and CL decreased by 50.14% and 87.75%, respectively. There was no significant change in the carotenoid content of PM as the pH of the SAR solution decreased, whereas the carotenoid content of CL decreased significantly more after the SAR pH 2.5 treatment (by 63.96%) than after the SAR pH 4.0 or 5.6 treatments.

**Figure 3:**
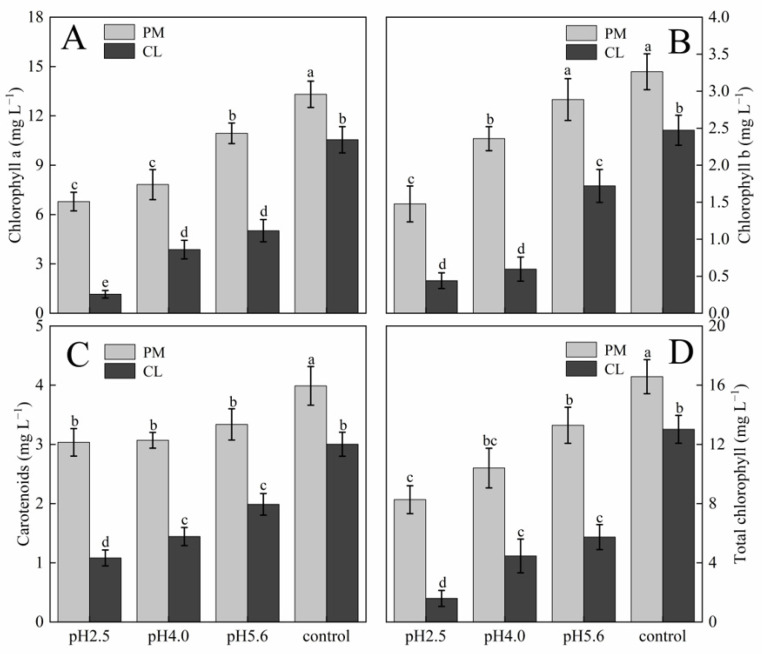
Photosynthetic pigment contents in PM and CL under SAR stress conditions: (**A**) Chlorophyll a. (**B**) Chlorophyll b. (**C**) Carotenoids. (**D**) Total chlorophyll. Each value is the mean ± SE (n = 3). Different lowercase letters indicates a significant difference at *p* < 0.05. Distinct OJIP curves were generated for both PM and CL treated with different SAR solutions. As the pH of the SAR solution decreased, the I–P phase of the OJIP curve decreased significantly (Figure 4). However, there was a difference between the two species. More specifically, the overall curve of PM decreased as the pH decreased, but the curve of CL revealed an increase in F_O_ as the pH decreased. Furthermore, in response to the decrease in pH, the OJIP curve gradually transformed into an OKJIP curve with an obvious K-band. In addition, following the SAR pH 2.5 and 4.0 treatments, the P point was eventually undetectable in the CL curve, whereas it was still detectable in the PM curve.

**Figure 4:**
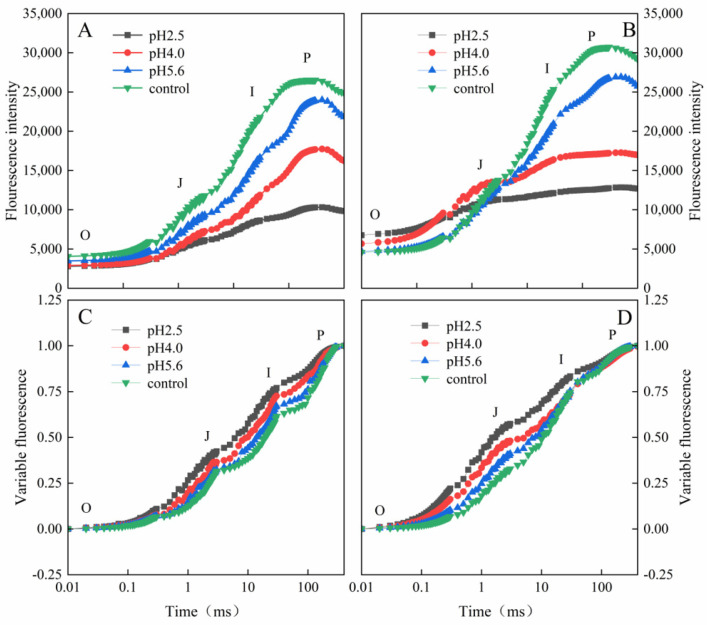
Chlorophyll a fluorescence kinetics curves and chlorophyll a fluorescence standardized curves for PM (**A**,**C**) and CL (**B**,**D**) under SAR stress conditions. Each curve represents the average data of five replicates. The letters O, J, I and P refer to the selected time points used by the JIP-test for the calculation of structural and functional parameters.

**Figure 5:**
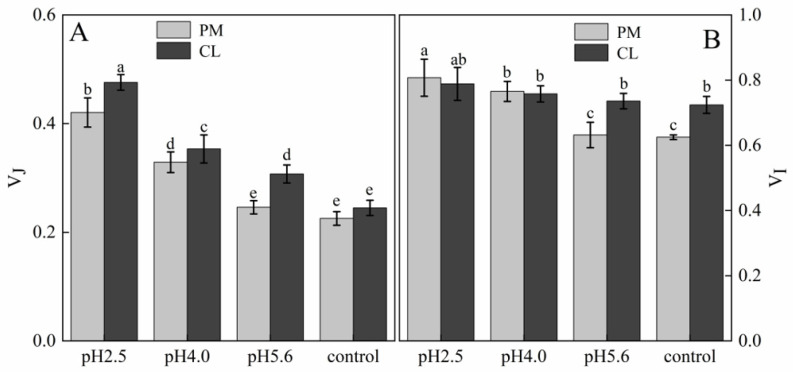
(**A**) Changes in the J point variable fluorescence of PM and CL under SAR stress conditions. (**B**) Changes in the I point variable fluorescence of PM and CL under SAR stress conditions. V_I_ = (F_I_ − F_O_)/(F_m_ − F_O_) and V_J_ = (F_J_ − F_O_)/(F_m_ − F_O_). Each value is the mean ± SE (n = 3). Different lowercase letters indicates a significant difference at *p* < 0.05. The fluorescence increase kinetics of PM and CL were normalized between the O point (0.05 ms) and the K point (0.3 ms) as follows: V_OK_ = (F_T_ − F_O_)/(F_K_ − F_O_). The K-band of both species was already higher than that of the control at pH 5.6. Further decreases in the pH resulted in significant increases in the K-band (Figure 6).

The normalized OJIP curves for both PM and CL indicated that the O–P points increased as the pH of the SAR solution decreased (relative to the control O–P points) (Figure 4). There were also increasing trends in V_J_ and V_I_ for both PM and CL (Figure 5). For PM, V_J_ and V_I_ did not change significantly at pH 5.6, but they increased significantly at pH 4.0. For CL, V_J_ increased significantly at pH 5.6.

**Figure 6:**
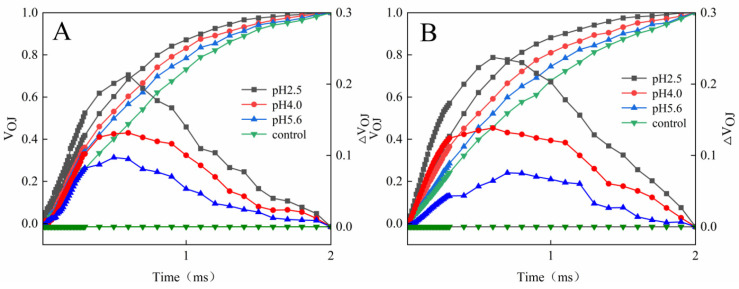
Changes in the K-bands of PM (**A**) and CL (**B**) in response to SAR stress. V_OJ_ = (F_T_ − F_O_)/(F_J_ − F_O_) and ΔV_OJ_ = V_OJ_(treatment) − V_OJ_(control).

**Figure 7:**
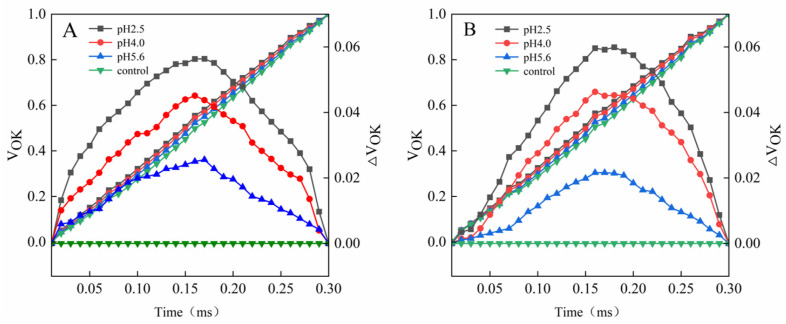
Changes in the L-bands of PM (**A**) and CL (**B**) in response to SAR stress. V_OK_ = (F_K_ − F_O_)/(F_m_ − F_O_) and ΔV_OK_ = V_OK_(treatment) − V_OK_(control).

The L-band was detected at pH 5.6 for both PM and CL. Further decreases in the pH were accompanied by significant increases in the L-band. Notably, the L-band of CL was higher than that of PM at pH 2.5 (Figure 7).

**Figure 8:**
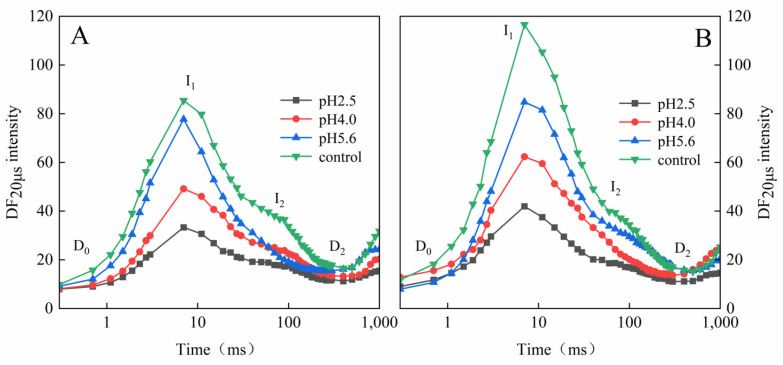
Delayed fluorescence induction curves of PM (**A**) and CL (**B**) under SAR stress conditions. Each curve represents the average data of five replicates. The peak at 3 ms was denoted as I_1_ and that at 100 ms as I_2_, and the initial minimum was labelled as D_0_ and the final plateau as D_2_.

The SAR treatments altered the amplitude and shape of the DF induction curves for both PM and CL (Figure 8A,B). The DF curves decreased as the pH of the SAR solution decreased, with the I_1_ peak decreasing faster than the I_2_ peak. As the pH value decreased, the I_2_:I_1_ ratio increased significantly, especially at pH 2.5 and 4.0. The I_2_:I_1_ ratio increased significantly at pH 5.6, but only for CL.

**Figure 9:**
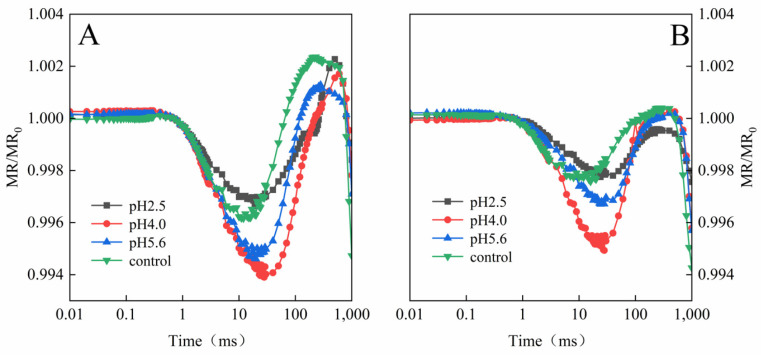
MR_820_ curves of PM (**A**) and CL (**B**) under SAR stress conditions. Each curve represents the average data of five replicates.

**Figure 10:**
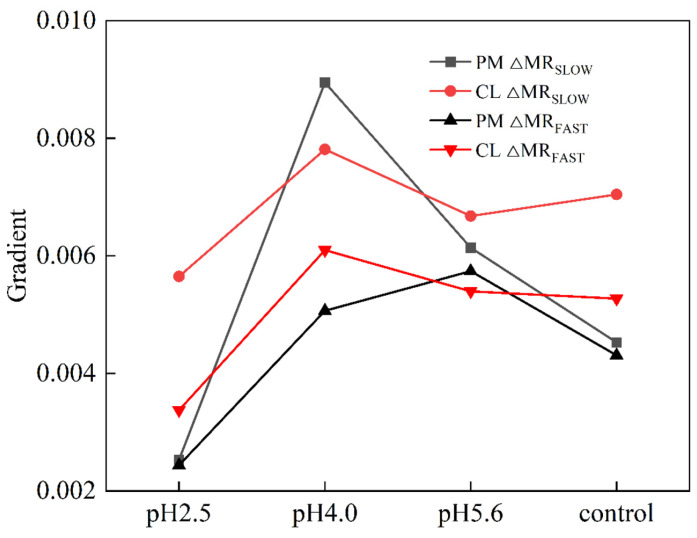
Gradient of the MR_820_ curves of PM and CL under SAR stress conditions. Each curve represents the average data of five replicates. ΔMR_SLOW_ = (MR_MAX_ − MR_MIN_)/MR_0_; ΔMR_FAST_ = (MR_0_ − MR_MIN_)/MR_0_.

The exposure to various levels of SAR stress led to significant changes to the MR/MR_0_ kinetics curves for both PM and CL, although the changes were clearer for CL (Figure 9). Decreases in the pH of the SAR solution shifted the lowest point of the MR/MR_0_ kinetics curve to a later time-point (Figure 9). The value of the maximum increase and decrease slope of MR/MR_820_ curves (ΔMR_SLOW_ and ΔMR_FAST_) in both PM and CL were increased and then decreased as the pH of the SAR solution increased (Figure 10). At pH 4.0, the value of ΔMR_SLOW_ and ΔMR_FAST_ in both PM and CL reach their maximum values. At pH 2.5, both the value of ΔMR_SLOW_ and ΔMR_FAST_ were lower for CL than for PM (Figure 10).

The SAR treatments decreased ABS/CS_m_, TR_O_/CS_m_, ET_O_/CS_m_, F_v_/F_m_, φE_O_, ψ_O_, PI_ABS_, and PI_TOTAL_ in both PM and CL, whereas they increased DI_O_/CS_m_ and φD_O_. However, there were differences between the two species. Specifically, decreases in pH resulted in increases and decreases in δR_O_ for PM and CL, respectively. Furthermore, significant decreases in various parameters, including ABS/CS_m_, TR_O_/CS_m_, F_v_/F_m_, and φE_O_, were detected at pH 4.0 and 5.6 for PM and CL, respectively.

## 3. Discussion

The harmful effects of SAR on plants are reflected by damaged leaf structures and decreased chlorophyll contents, which have inhibitory effects on photosynthesis [6]. Additionally, SAR-induced damages to the stomata of leaves adversely affect plant respiration and other physiological activities [7]. The chlorophyll content, Pn, and Gs decreased significantly in the PM and CL leaves after the SAR pH 4.0 and 2.5 treatments, whereas Ci increased as the pH decreased, implying that the decrease in photosynthetic performance in PM and CL was unrelated to stomatal factors.

The main phases of the OJIP curve in response to SAR stress differed between the two analyzed tree species. Specifically, the I–P phase decreased more significantly for CL than for PM. The comparison with CL indicated that the fluorescence peak (F_P_) increased more in PM as the pH decreased and the I–P phase decreased more under low-pH conditions (Figure 4A,B). The increase in prompt chlorophyll a fluorescence during the J–I phase reflects the extent of the conversion from QA^−^ to QA, whereas the decrease in the PQ pool leads to a decrease in the primary electron acceptor QA levels because of the limited induction of PQ reduction [29]. The I point represents the equilibrium between the reduction of the PQ pool by the electrons of PSII and the re-oxidation of the PQ pool, whereas the P point represents the completion of the reduction of all electron acceptors in PSII and PSI [30]. In the current study, the I–P phase, which is related to the PSI activity, indicated that the low-pH SAR treatment inhibited the electron transfer process in PM and CL. More specifically, blocking the transfer of electrons on the PSII acceptor side led to affecting the reduction and re-oxidation of the PQ pool. Furthermore, under low-pH SAR stress conditions, the OJIP curve gradually changes to the OKJIP curve, with the K point detected at approximately 0.3 ms. The appearance of the K point is mainly due to the inhibition of H_2_O splitting and the damage to the OEC caused by the partial inhibition of QA, indicative of further damages to PSII.

The relative variable fluorescence (V_J_) at 2 ms for unconnected PSII units is equal to the fraction of closed reaction centers (RCs) at the J-step expressed as a proportion of the total number of the RCs that can be closed [31,32]. A previous study showed V_I_ reflects the capacity of PS I and its receptor quinone to oxidize and reduce. Under stress conditions, changes in V_I_ reportedly represent decreases in the acceptance of electrons by PQ, leading to an inhibited transfer of electrons from QA to QB [32]. In the present study, the SAR treatments of PM and CL had detrimental effects on the ability to accept electrons from PQ, resulting in the accumulation of QA and the inability to transfer electrons from QA to QB (Figure 5).

A damaged OEC (commonly indicated by V_OK_) obstructs the transfer of electrons from the primary electron donor to the secondary electron donor and leads to an imbalanced electron flow between the donor and receptor sides [33]. The ΔV_OK_ value represents the standardized V_OK_ value. The V_OJ_ value indicates that the rate of electrons captured by the pigments in the PS II RCs exceeds the rate of the exchange between QA^−^ at the QB site and the oxidized PQ pool [33]. The ΔV_OJ_ value represents the standardized V_OJ_ value. In this study, a decrease in the pH of the SAR solution negatively affected both V_OK_ and V_OJ_. As the pH decreased, ΔV_OK_ and ΔV_OJ_ increased, but there were significant differences between CL and PM at pH 2.5 (Figure 6 and Figure 7).

When electrons are transferred to the PSII RCs, the associated DF is caused by the reverse flow of electrons, leading to charge recombination and the subsequent re-population of the PSII antenna chlorophylls [19,24]. The intensity of DF directly depends on the rate of the reverse electron transfer reaction in the PSII RCs [22,34]. The shape of the induced DF curve varies among sample types and physiological states [19,24,35], but it also depends on DF kinetics [36]. The induced DF curve was constructed using DF signals recorded at the same decay time-points in multiple DF decay curves (Figure 8). In the DF curve, the peak value at 3 ms was designated as I_1_, the peak value at approximately 100 ms was designated as I_2_, the initial minimum value was designated as D_0_, and the final plateau was designated as D_2_ [37]. After the SAR treatment, the I_1_ and I_2_ decreased as the pH decreased, with faster decreases in the I_1_ peak than in the I_2_ peak. This indicated that the number of PSII RCs and the PSII donor-side and acceptor-side electron transfer capacity were decreased by SAR. 

A typical 820 nm kinetics curve includes a decreasing phase followed by an increasing phase. The decrease reflects the oxidation of PSI and PC, whereas the subsequent increase is indicative of the reduction of PSI and PC. The PC and PSI reduction rates are equal to their oxidation rates at the lowest point of the decreasing phase [19]. The 820 nm optical reflectance curve is commonly used to represent the effects of different treatments on PSI [20]. The MR/MR_0_ value was calculated, where MR_0_ is the value at the onset of the actinic illumination (0.7 ms; the first reliable MR measurement). An increase in MR/MR_0_ indicates a decrease in the concentration of the oxidized states of plastocyanin (PC^+^) and the PSI RCs (P700^+^), which is due to the reduction of PC^+^ and P700^+^ [19]. The accumulation of P700^+^ and PC^+^ increases 820 nm absorption, resulting in a decreased fast phase (ΔMR_FAST_ = (MR_0_ − MR_MIN_)/MR_0_.). Subsequently, electrons coming from the PSII RCs arrive at P700^+^ and PC^+^ and re-reduce them, causing the decrease in ΔMR_FAST_ to slow down. Once the re-reduction rate is faster than the oxidation rate, ΔMR_FAST_ begins to increase (slow phase, ΔMR_SLOW_ = (MR_MAX_ − MR_MIN_)/MR_0_) [38]. Decreases in the pH of the SAR solution decreased the lowest point of the MR curve and shifted the lowest point to a later time-point (Figure 9). At pH2.5, the severe damage destroyed P700^+^ and PC^+^, which results in low gradients in the ΔMR_FAST_ and ΔMR_SLOW_ (Figure 10). (Figure 10), indicative of the severity of the SAR stress. Notably, the decrease in the slope was greater for CL than for PM, implying PM is more tolerant to SAR stress than CL.

**Figure 11:**
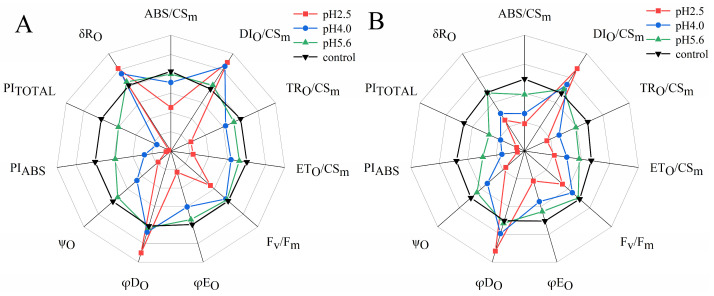
Radar maps of the JIP test parameters derived from the OJIP transient curves of the chlorophyll a fluorescence of PM (**A**) and CL (**B**) under SAR stress conditions. The value for the comparison was set to 1 for each parameter.

According to earlier research, ABS/CS_m_, TR_O_/CS_m_, ET_O_/CS_m_, and DI_O_/CS_m_ reflect the efficiency of each index per cross-section unit at t = t_Fm_ [39]. In the present study, the exposure to SAR stress significantly decreased ABS/CS_m_, TR_O_/CS_m_, and ET_O_/CS_m_, but increased DI_O_/CS_m_ (Figure 11). The decrease in ABS/CS_m_ was due to the deactivation of the RCs caused by SAR as well as the destruction of the antenna pigment structure, which decreased the captured light energy, the excitation energy, and reduction energy of the RCs while also altering the transfer of electrons. The increase in DI_O_/CS_m_ suggests that the SAR treatments activated a defense mechanism through which excess excitation energy in the leaves was dissipated quickly to limit damages. Additionally, φD_O,_ φE_O_, and Ψ_O_ are important quantum indicators for the electron transport chain (Figure 11). Furthermore, δR_O_ represents the efficiency with which electrons are transferred from the reduction system to the PSI electron acceptor side; it is also a photosynthetic performance index that is based on light absorption (PI_ABS_), making it an important parameter for studying the photosynthetic status of plants. In this study, as well as in earlier studies, PI_TOTAL_ served as the comprehensive photosynthetic performance index (Figure 11) [21]. F_v_/F_m_ is related to the degree of photoinhibition [40]. F_v_/F_m_ dropped significantly in both PM and CL at pH4.0 and pH2.5, which means that a low-pH SAR causes photoinhibition. We also found that F_v_/F_m_ of CL dropped more from pH5.6 to pH4.0 than PM; thus, CL is less tolerant to SAR. Under SAR stress conditions, F_v_/F_m_, φE_O_, Ψ_O_, PI_ABS_, and PI_TOTAL_ decreased significantly for both PM and CL, which was in contrast to the significant increase in φD_O_. For PM, the change in δR_O_ in response to decreases in pH indicates that PSI was more involved in the cyclic transfer of electrons than in the linear transfer of electrons. Increases in cyclic electron transfer are critical for minimizing SAR-induced damages to the photosynthetic system. 

## 4. Materials and Methods

### 4.1. Plant Materials and Treatments

Two-year-old PM and CL seedlings that were similar in height were used in this study. The seedlings were obtained from Jiujiang Decheng Landscape Greening Co., Ltd. (Jiujiang, China) and then grown in plastic tubes (35 cm tall, 25 cm upper diameter) filled with a 1:1:3 (*v*/*v*) mixture of vermiculite, perlite, and peat soil. Plants were grown outdoors at Jiyang College in Zhuji, Zhejiang, China. Approximately 3 months later, 48 PM and CL seedlings were transferred to two growth chambers, respectively. The settings for both growth chambers were as follows: a 14 h photoperiod with a photosynthetic photon flux density (PPFD) of approximately 600 μmol m^−2^ s^−1^; a relative humidity of approximately 50%; and a 25 °C (day)/20 °C (night) cycle. Fertilizer-containing water and half-strength Hoagland solution were applied to the plants once per week.

H_2_SO_4_ and HNO_3_ (concentration of 98%) were used to prepare the acid rain stock solution (molar ratio of SO_4_^2−^ to NO_3_^−^ of 2.4:1 and pH 1.0) based on the chemical composition of the acid rain in Zhejiang province over the previous 3 years. The stock solution was diluted with deionized water to prepare SAR solutions with varying pH values (2.5, 4.0, and 5.6) that corresponded to different SAR stress levels. Deionized water (pH 6.8) was used for the control treatment. After two weeks of cultivation in the growth chamber, SAR treatments were imposed by randomly allocating seedlings to four groups in each chamber. Each seedling (from top to bottom) was sprayed uniformly with the 50 mL SAR solutions or deionized water at 9:30 a.m. once every 2 weeks. After spraying the acid rain for 60 days (stress period), all the measurements were performed. 

### 4.2. Gas Exchange Measurements

The LI-6400 portable photosynthesis system (LI-COR, Lincoln, NE, USA) was used to measure the gas exchange parameters of PM and CL. Specifically, the third pair of intact functional leaves from the top of the seedling stem were analyzed, with three replicates per leaf. The measurements were conducted using the built-in red–blue light source, with a PPFD of 1200 µmol m^−2^ s^−1^, a CO_2_ concentration of 400 µmol m^−2^ s^−1^, a leaf chamber temperature of 25 °C, and a flow rate of 300 mmol s^−1^. 

### 4.3. Determination of the Chlorophyll Content

The third pair of intact functional leaves from the top of the seedling stem were collected for the determination of the chlorophyll content as described by Lichtenthaler [41].

Chlorophyll a content (mg L^−1^): C_a_ = 13.95 A_665_ − 6.88 A_649_.

Chlorophyll b content (mg L^−1^): C_b_ = 24.96A_649_ − 7.32 A_665_.

Total chlorophyll content (mg L^−1^): C_a+b_ = C_a_ + C_b_.

Carotenoids content (mg L^−1^): C_c_ = (1000A_470_ − 3.27C_a_ − 104C_b_)/229.

### 4.4. Simultaneous Measurement of PF, DF, and MR Kinetics

The third pair of intact functional leaves from the top of the seedling stem was selected and maintained in darkness for 30 min. The chlorophyll fluorescence parameters were measured using M-PEA (Hannsatech Instruments Ltd., King’s Lynn, UK). A red-light pulse (650 nm, 3500 μmol m^−2^ s^−1^) resulted in an increase in the fluorescence curve. In terms of the chlorophyll fluorescence curve, O represents the initial fluorescence level; K (0.3 ms), J (2–3 ms), and I (30 ms) represent the intermediate fluorescence levels; and P (500 ms) represents the peak fluorescence level. We also used the JIP test to analyze the OJIP fluorescence transients [39,42]. The JIP test defines the maximum energy flux of absorption (ABS), trapping (TR), electron transport (ET), and dissipation (DI) in the excitation cross-section (CS) cascade. This test is based on the basic theory of energy flow through the thylakoid membrane and the total energy flow from the light-harvesting complex (i.e., energy flux ratio).

The data were analyzed using the JIP test [42]. Several basic data were collected in this study, including F_O_ (fluorescence intensity at 0.02 ms), F_L_ (fluorescence intensity at 0.15 ms), F_K_ (fluorescence intensity at 0.3 ms), F_J_ (fluorescence intensity at 2 ms), F_I_ (fluorescence intensity at 30 ms), and F_m_ (maximum fluorescence intensity, which is equal to F_P_). To analyze the electron transport chain activity, the following relative fluorescence parameters were calculated via the double-normalization of the moment chlorophyll fluorescence values against the end points in different time intervals in the OJIP part of the transient: OP, OK, OJ, OI, and IP; V_T_, the relative variable fluorescence at time T, which is calculated using the formula V_T_ = (F_T_ − F_O_)/(F_m_ − F_O_); W_OK_, the ratio of the variable fluorescence (F_T_ − F_O_) to the amplitude (F_K_ − F_O_), which is used to represent the L-band; and W_OJ_, which is calculated using the formula W_OJ_ = (F_T_ − F_O_)/(F_J_ − F_O_) and used to represent the K-band.

The parameters that refer to time 0 include the following: the maximum quantum yield of PSII primary photochemistry (φP_O_ = TR_O_/ABS = F_v_/F_m_); the efficiency with which a trapped exciton moves an electron into the electron transport chain beyond the reduced QA form (QA^−^) (ψE_O_ = ET_O_/TR_O_); the quantum yield of electron transport (φE_O_ = ET_O_/ABS); the average fraction of open PSII RCs in the time interval between 0 and t_Fm_ (S_m_/t_Fm_); the fraction of active PSII RCs per CS (RC/CS); the absorption flux per CS (ABS/CS); the trapped energy flux per CS (TR_O_/CS); the electron transport flux per CS (ET_O_/CS); the absorption flux per RC (ABS/RC); the quantum yield for the reduction of the end electron acceptors on the PSI acceptor side (φR_O_ = RE_O_/ABS); and the probability that an electron is transported from the reduced intersystem electron acceptors to the final electron acceptors of PSI [δR_O_ = RE_O_/ET_O_ = (1 − V_I_)/(1 − V_J_)].

### 4.5. Data Analysis

All experiments were repeated at least three times. All values are expressed herein as the mean ± standard deviation. Statistical analysis was performed using SPSS 20.0 (IBM, Armonk, NY, USA). Differences among treatments were analyzed by least significance difference (LSD) test at *p* < 0.05.

## 5. Conclusions

In this study, Pn and Gs decreased significantly in the PM and CL leaves after low-pH SAR treatments, whereas Ci increased as the pH decreased, implying that the decrease in photosynthetic performance in PM and CL was unrelated to stomatal factors. The decrease in the I–P phase was due to the inhibited transfer of electrons on the electron acceptor side of PSI. The increase in PF transient during the J–I phase, which was indicated by V_J_ and V_I_, reflected the extensive transfer of electrons from PSI to the PQ pool. However, the re-oxidation of the PQ pool was insufficient, leading to the accumulation of QA^−^. The appearance of the L-band and K-band demonstrates that SAR stress interferes with the transfer of electrons on the donor and acceptor sides, thereby decreasing the transfer of energy to the active RCs of PSII. This results in the dissociation of the PSII antenna complex from the PSII core proteins, which adversely affects the OEC and destabilizes the PSII system. After the SAR treatment, the I_1_ and I_2_ in DF curves decreased as the pH decreased, with faster decreases in the I_1_ peak than in the I_2_ peak. This indicated that the number of PSII RCs and the PSII donor-side and acceptor-side electron transfer capacity were decreased by SAR. Analysis of the MR_820_ kinetics showed the severe damage destroyed P700^+^ and PC^+^, which results in low gradients in the ΔMR_FAST_ and ΔMR_SLOW_. In terms of the JIP test parameters, ABS/CS_m_, TR_O_/CS_m_, ET_O_/CS_m_, F_v_/F_m_, φE_O_, and Ψ_O_ decreased significantly after the SAR treatments, resulting in decreases in PI_ABS_ and PI_TOTAL_. Furthermore, the SAR-induced increases in both DI_O_/CS_m_ and φD_O_ protected PSII from membrane photo-oxidation. Decreases in pH caused δR_O_ to increase, but only for PM. In conclusion, the decrease in chlorophyll contents and the damages to the OEC in PM and CL disrupted the transfer of electrons on the donor and acceptor sides of PSII, which inhibited Pn and suppressed the growth of PM and CL. However, PM was more tolerant to SAR than CL, which may be related to the observed difference in δR_O_ between the two tree species. Thus, the physiological functions of CL may already be damaged at pH 5.6, while those of PM are generally unaffected until the pH decreases to 4.0.

## Data Availability

The data presented in this study are available upon request from the corresponding author. The data are not publicly available due to privacy.
